# Community pharmacists’ perceptions on providing fall prevention services: a mixed-methods study

**DOI:** 10.1007/s11096-021-01277-4

**Published:** 2021-06-13

**Authors:** Marle Gemmeke, Ellen S. Koster, Eline A. Rodijk, Katja Taxis, Marcel L. Bouvy

**Affiliations:** 1 Division of Pharmacoepidemiology and Clinical Pharmacology, Utrecht Institute for Pharmaceutical Sciences (UIPS), Faculty of Science, Utrecht University, PO Box 80082, 3508 TB Utrecht, The Netherlands; 2 Department of Pharmacotherapy, Pharmacoepidemiology and Pharmacoeconomics (PTEE), Faculty of Science and Engineering, Groningen Research Institute of Pharmacy, University of Groningen, Groningen, The Netherlands

**Keywords:** Accidental falls, Community pharmacy services, Decision making, Deprescriptions, Pharmacists

## Abstract

*Background* Pharmacists may contribute to fall prevention particularly by identifying and deprescribing fall risk-increasing drugs (FRIDs) in patients with high fall risk. *Objective* To assess community pharmacists’ perceptions on providing fall prevention services, and to identify their barriers and facilitators in offering these fall prevention services including deprescribing of FRIDs. *Setting* A mixed-methods study was conducted with Dutch pharmacists. *Method* Quantitative (ranking statements on a Likert scale, survey) and qualitative data (semi-structured interviews) were collected. Out of 466 pharmacists who were invited to participate, 313 Dutch pharmacists ranked statements, about providing fall prevention, that were presented during a lecture, and 205 completed a survey. To explore pharmacists’ perceptions in-depth, 16 were interviewed. Quantitative data were analysed using descriptive statistics. All interviews were audiotaped and transcribed verbatim. The capability opportunity motivation-behaviour model was applied to interpret and analyse the findings of qualitative data. *Main outcome measure* Community pharmacists’ views on providing fall prevention. *Results* Pharmacists stated that they were motivated to provide fall prevention. They believed they were capable of providing fall prevention by FRID deprescribing. They perceived limited opportunities to contribute. Major barriers included insufficient multidisciplinary collaboration, patient unwillingness to deprescribe FRIDs, and lack of time. Facilitators included goal-setting behaviour, financial compensation, and skilled communication. *Conclusion* Despite the complex decision-making process in medication-related fall prevention, community pharmacists are motivated and feel capable of providing fall prevention. Opportunities for pharmacists to provide fall prevention services should be enhanced, for example by implementing multidisciplinary agreements.

**Supplementary Information:**

The online version contains supplementary material available at 10.1007/s11096-021-01277-4.

## Impacts on practice

- Multidisciplinary agreements defining community pharmacists’ tasks would support pharmacists to collaborate in fall prevention.
- Financial compensation for the provision of fall prevention services could motivate community pharmacists to provide fall prevention.
- The development of clinical decision tools could facilitate FRID deprescribing by community pharmacists.

## Introduction

Worldwide, one third of community-dwelling persons aged 65 years and older falls at least annually [1, 2]. The number of falls is growing due to increased life expectancy and aging of the general population [2]. Serious consequences of falls include traumatic brain injury, fractures, functional decline, decreased quality of life, and death [3]. Falling is a multifactorial problem caused by many underlying factors, such as mobility and vision problems [4], and medication use has also often been associated with increased fall risk. For example, fall risk-increasing drugs (FRIDs) include cardiovascular and psychotropic drugs because of their potential to cause fall-related side effects [5–7]. Hence, prevention of falls is gaining attention among community pharmacists [8–10].

Community pharmacists have frequent contact with patients and may have the opportunity to identify those with high fall risk. This is because pharmacists may recognize medication-related falls and could therefore play an important role in fall prevention [11, 12]. Deprescribing FRIDs may be effective in reducing falls [13, 14]; so far, community pharmacists have contributed to fall prevention by performing medication reviews to reduce side effects as dizziness and sedation [15, 16]. Pharmacists could also refer patients to other healthcare providers, for example general practitioners (GPs), physiotherapists, and home care nurses [17]. Finally, like other healthcare providers, pharmacists can provide general advice on fall prevention, for example lifestyle recommendations [4, 8].

The Medical Research Council Framework guides the development and evaluation of complex interventions, and consists of four phases: development, feasibility/piloting, evaluation, and implementation. Understanding the changes in processes of an intervention is a key element of implementation [18]. Several barriers repeatedly arose during implementation of fall prevention programmes in different healthcare settings. For example, older persons are often not aware of their fall risk and therefore not engaged in fall prevention. Furthermore, lack of time of healthcare professionals is an important barrier [19–21]. Awareness about the importance of fall prevention varies among healthcare providers [22]. Due to the multicausality of falls, decision-making regarding how to prevent falls is often complex; therefore, fall prevention benefits from a multidisciplinary approach. In practice, organizing well-tuned co-operative fall prevention care is challenging, and a lack of guidance and training hinders healthcare providers’ provision of fall prevention [20, 21]. Fall prevention is consequently less integrated into daily routines than other preventive measures, such as cancer screenings [20].

In Ohio, most pharmacists believed they can contribute to safe FRID use in patients with high fall risk [23]. In another previous study the majority of community pharmacists in Montreal thought they should conduct medication reviews with patients with high fall risk, but only a minority reported actually being involved. Likewise, pharmacists in this study were less involved than they wished in other fall prevention services, including fall risk assessment, provision of information/recommendations to patients, and referral to fall prevention programs [24]. Therefore, despite all the efforts, few community pharmacist-led fall prevention services are implemented in practice thus far and the barriers and facilitators for implementation remain unclear. The current state of community pharmacist-led services of fall prevention should, therefore, be examined including pharmacist’s thoughts about barriers and facilitators. Such information is the foundation for initiating behavioural change among pharmacists in practice, in order to provide fall prevention care, and it is needed to implement pharmacist-led fall prevention services in the future [25].

## Aim of the study

In this mixed-methods study, we aimed (1) to assess community pharmacists’ perceptions on providing fall prevention services and (2) to identify the barriers and facilitators in providing these fall prevention services, including the deprescribing of FRIDs.

## Ethics approval

The study protocol was approved by the Institutional Review Board of the Division of Pharmacoepidemiology and Clinical Pharmacology, Utrecht University (reference number UPF2002).

## Method

### Design, setting and participants

A mixed methods study was conducted combining quantitative and qualitative data collection methods. Participants were invited to participate in this study during five regional meetings of the Royal Dutch Pharmacists Association (Koninklijke Nederlandse Maatschappij ter bevordering der Pharmacie, [KNMP]) which were organized to educate and inform community pharmacists about fall prevention. The regional meetings were part of the routine educational programme offered by the KNMP for their members in all five regions spread across the Netherlands. Pharmacists enrolled voluntarily in the meetings, which all were held in February 2020.

#### Quantitative and qualitative data

**Fig. 1:**
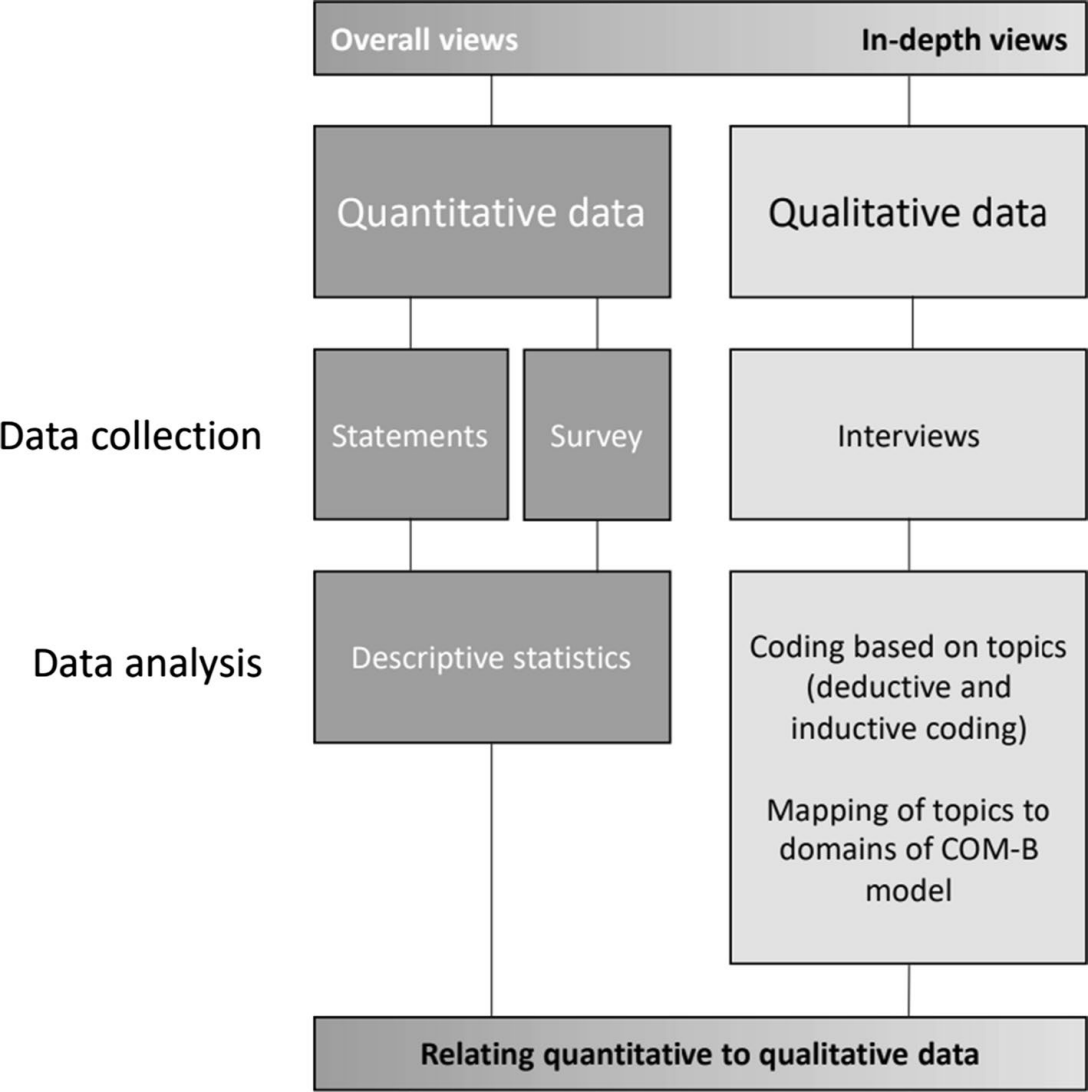
Application of quantitative methods (statement rankings and survey) and a qualitative method (interviews) to investigate the overall and in-depth perspectives of pharmacists. The capability opportunity motivation-behaviour (COM-B) model was applied to qualitative data. Quantitative data and qualitative data were related to each other by linking findings by means of the topics. *COM-B* capability opportunity motivation-behaviour model

Pharmacists’ overall perspectives were primarily investigated by quantitative methods: statement rankings during an interactive lecture, and a survey. To investigate their in-depth perspectives, qualitative interviews were conducted. Figure 1 summarizes how quantitative and qualitative data were collected and analyses, as described in the sections below.

The results were reported according to the consolidated criteria for reporting qualitative research (COREQ) guidelines (see Supplementary Information S1) [26].

### Quantitative data collection

#### Statements

During the five regional meetings, pharmacists participating in the lectures were asked to rate nine statements on the fall prevention activities of community pharmacists and their need for further implementation of fall prevention on a Likert scale from 1 (totally disagree) to 10 (totally agree) (see Supplementary Information S2 for the content of statements). Examples of statements were as follows: “I have enough knowledge to recognize FRIDs” and “At the moment, I contribute to fall prevention”. Through discussion, the research team developed the statements, which were based on literature findings, until they all agreed on sufficient applicability [9, 10, 17]. The presentation software Mentimeter (www.mentimeter.com) was employed to display the statements and record the responses. Pharmacists in the audience used their smartphones to rank the statements, and they were asked for permission to use the responses for research purposes after the lecture.

#### Survey

After the lecture pharmacists were immediately invited to complete a paper-based survey (see Supplementary Information S2 for the content of the survey). The survey was in Dutch and comprised of 26 questions. The topics were: current fall prevention activities of pharmacists, fall risk assessment during medication review, needs for assistance for further implementation of fall prevention, and needs for a guideline to deprescribe FRIDs. Topics were based on literature findings, and through discussion, the research team developed the survey until they all agreed on sufficient applicability [9, 10, 17]. The survey also collected background information, including age, gender, and years of work experience. The types of questions varied: statements (using a Likert scale from 1 to 5), open sections, and multiple choice questions. All responses were processed anonymously.

### Qualitative data collection

In the survey, pharmacists could indicate their interest in an interview with a master student-researcher (ER) to explain their perceptions on pharmacist-led fall prevention. By means of interviewing, we obtained in-depth information regarding the community pharmacists’ perspective on fall prevention services, including barriers and facilitators in establishing such services in practice. The interviews were held between April 2020 and June 2020 by telephone. All participants provided verbal informed consent, and all interviews were audio recorded. The interviews were guided by a topic list that included the following topics: knowledge of FRIDs, deprescribing, multidisciplinary collaboration, and helpful tools for deprescribing (see Supplementary Information S2). Topics were identified based on literature, themes that arose out of the survey, and themes that emerged during short talks with community pharmacists about fall prevention during the regional meetings of the KNMP [9, 10, 17]. The topic list was evaluated after the first three interviews, and only a few questions were slightly adjusted. Data saturation was determined after 16 interviews on the basis of whether new findings emerged in the last three interviews.

### Data management and analysis

#### Quantitative data

Participants who did not give permission to use their answers to the statements ranked during the presentation were excluded from the analyses. Answers from written surveys were entered in Microsoft Office Excel® 2019. Then, descriptive statistics, including frequencies, medians and interquartile range were calculated. All analyses were performed using R version 3.6.3 software.

#### Qualitative data

All audio recordings of the interviews were transcribed verbatim and imported into NVivo version 12 software. All interviews were anonymized by replacing participants’ names with participant numbers. The audio recordings and transcripts were stored on a virtual protected server only accessible to the research team.

The capability opportunity motivation-behaviour (COM-B) model was applied to analyse and interpret the qualitative data [27, 28]. The COM-B model is a widely used behavioural change theory and therefore a suitable framework to identify needs to change [29]. The COM-B model has been used to describe healthcare providers’ dependencies to express a desired behaviour [27, 28]. According to the COM-B system, pharmacists will provide fall prevention when the following conditions are met:

- Capability: pharmacists need to have the knowledge and skills to provide fall prevention care and deprescribe FRIDs.
- Opportunity: pharmacists need to have time, and knowledge about their patients’ fall risks, and the (deprescribing) activities should be affordable.
- Motivation: pharmacists should be motivated to implement fall prevention care and the deprescribing of FRIDs in daily practice [27, 28].

The interviews were coded by a postgraduate student researcher (MG) and reviewed by an experienced postgraduate researcher (EK). A topic list, prepared in advance, was used to guide the coding (deductive coding). During the coding process, a number of additional topics were identified (inductive coding), and possible discrepancies were resolved through discussion. Pharmacists quotations for implementing fall prevention care were deductively linked to the related domains of the COM-B model by one researcher (MG) and checked by two researchers (EK, MB). Possible discrepancies were resolved through discussion.

## Results

### Background characteristics

**Fig. 2:**
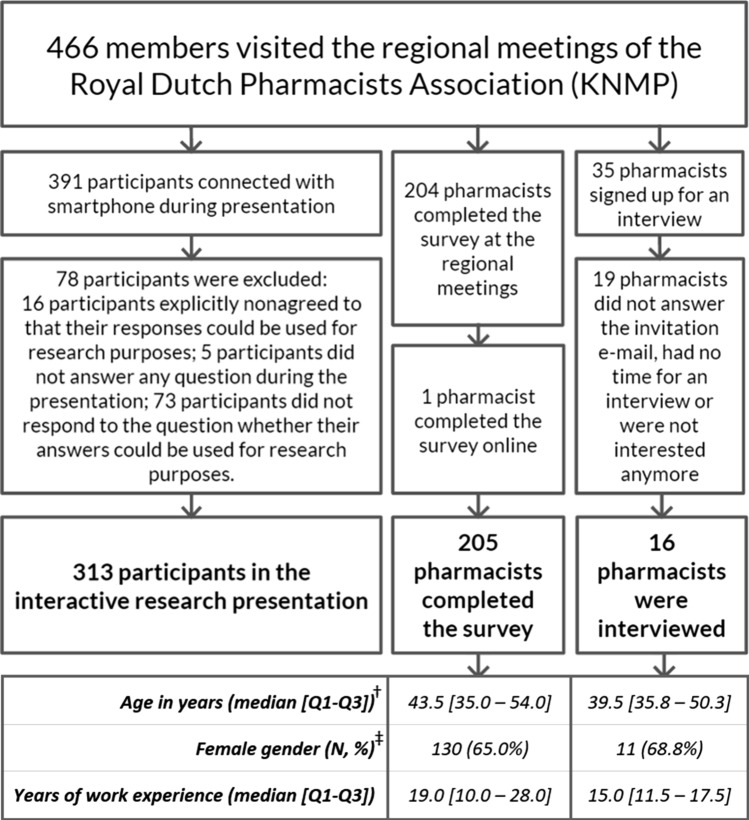
Flowchart and background characteristics of responders to the statements during the interactive research presentation, survey and inclusion of pharmacists in interviews. ^†^Five pharmacists did not share background characteristics, but completed the survey. ^‡^One pharmacist did not share his/her years of working experience. *Q1* first quartile, *Q3* third quartile, *N* number

The five regional meetings were attended by 466 members of the KNMP and all of them were invited to participate. As illustrated in Fig. 2, data from 313 participants who responded during the lecture were analysed.

In total, 205 pharmacists completed the survey and 16 of them participated in a telephone interview (Fig. 2). Most were female (65.0% and 68.8% in the survey and interviews, respectively). The median work experience was 19 years [Q1–Q3: 10.0–28.0 years] for the survey and 15 years [Q1–Q3: 11.5–17.5 years] for the interviews. The duration of the interviews varied between 20 and 35 min.

### Overall perspectives (quantitative data)

Tables 1 and 2 show pharmacists’ responses to the statements in the interactive lecture and survey, as clustered and described below.

**Table 1:** Pharmacists’ responses to statements during the interactive lecture and in the survey

Interactive lecture (statement rankings on a Likert scale *from completely disagree (0) to completely agree (10))*			Survey (statement rankings on a Likert scale *from disagree (1) to agree (5))*		
Number	Statement	Median [Q1–Q3]	Number	Statement	Median [Q1–Q3]
S1	Community pharmacists can contribute to fall prevention	8 [7–10]	S1	I proactively ask patients about fall history (at the counter or on a telephone call)	2 [2, 3]
S2	I have enough knowledge to recognize FRIDs	8 [7–9]	S2	The pharmacy technicians proactively ask patients about fall history	2 [1, 2]
S3	I have the capabilities to recognize patients with high fall risk	6 [5–7]	S3	I experience difficulties recognizing patients with high fall risk	3 [2–4]
S4	At the moment, I contribute to fall prevention	4 [1–5]	S4	I experience difficulties starting a conversation with patients about the effects of their medication use on their fall risk	2 [2, 3]
S5	I discuss fall prevention at medication reviews	8 [6–10]	S5	I ask about fall history when I perform a medication review	4 [3–5]
S6	Beyond medication reviews, I discuss fall prevention	2 [0–5]	S6	When I perform a medication review, I suggest medication modifications if I know the patient has fall experiences	4 [3–5]
S7	I have enough time to organize fall prevention care	4 [2–6]	S7	I am going to spend more time and attention on fall prevention in my daily practice	4 [3, 4]
S8	Recognizing patients with fall risk belongs to one of the tasks of community pharmacists	6 [5–8]	S8	I discuss with patients their risk factors for falling	2 [1–3]
S9	Fall prevention care belongs to tasks of community pharmacists	7 [5–8]	S9	The pharmacy technicians discuss patients’ risk factors for falling with them	2 [1, 2]
			S10	I need a guideline that supports me with deprescribing FRIDs	4 [3, 4]
			S11	A guideline that supports me to deprescribe FRIDs is not going to help me, because deprescribing should be tailored to individual patient circumstances	3 [2–4]

**Table 2:** Findings of survey questions related to multidisciplinary agreements about fall prevention and pharmacists’ needs for contributing to fall prevention

Question	Answer	N (%)
Do you have multidisciplinary agreements about fall prevention? N = 205	Yes	43 (21%)
	No	146 (71%)
	No response	16 (8%)
If you have multidisciplinary agreements about fall prevention, with whom? N = 43	General practitioner	39 (91%)
	Physiotherapist	19 (44%)
	Home care	18 (42%)
	Nursing home physician	11 (26%)
	Dietician	5 (12%)
	Geriatrician	3 (7%)
	Other†	6 (14%)
What are your needs to be able to do more in fall prevention? N = 192	Multidisciplinary collaboration	140 (73%)
	Reimbursement	137 (71%)
	Time	128 (67%)
	Training for pharmacist technicians	120 (63%)
	Patient information material	112 (58%)
	A guideline to deprescribe FRIDs	97 (51%)
	More knowledge/training	70 (36%)

*N* number, *FRID* fall risk-increasing drug

^†^Psychologist, community project/social team, occupational therapist, optician and district nurse were mentioned in the survey

#### Knowledge and skills

Community pharmacists believe they could contribute to fall prevention. The survey results indicate that most pharmacists believe they are able to identify patients with high fall risk, but some have experienced difficulties with this. Furthermore, pharmacists reported that they already suggest medication modifications when patients report falls during medication reviews. Pharmacists believe they have sufficient knowledge to recognize FRIDs. In the survey, only 36% of the pharmacists reported a need for more knowledge or training. However, pharmacists mentioned needing a guideline for the deprescribing of FRIDs. On the other hand, because of the complexity of deprescribing, they revealed doubt about whether this would help them.

#### Collaboration

In the survey, pharmacists expressed the need for increased multidisciplinary collaboration in fall prevention (73%). Collaboration with GPs, home care providers, physiotherapists and geriatricians was found to be especially important. Most pharmacists (71%) did not have specific multidisciplinary agreements about fall prevention yet. For those who had multidisciplinary agreements, these were most often concluded with GPs (91%), followed by physiotherapists (44%) and home care nurses (42%). Based on the findings, collaboration with GPs seems to be best-organized, since pharmacists reported discussing fall prevention mainly in collaborative medication reviews with GPs. Fall prevention was rarely discussed outside the scope of medication reviews.

#### Time and reimbursement

Pharmacist believe that community pharmacists are responsible for fall prevention, and they hence reported that they aim to spend more time and attention on fall prevention. In the survey, the majority of pharmacists (67%) reported not having enough time for fall prevention activities. Moreover, 71% of pharmacists reported that they need financial compensation for fall prevention in order to provide certain care.

#### Identification of patients

Fall prevention starts with the identification of patients at risk of falling. To a lesser extent, pharmacists also consider that the identification of patients with high fall risk belongs to be a task of community pharmacists. The survey showed that most pharmacists ask patients about fall history in medication reviews, but they less frequently, proactively ask patients about fall history during other regular encounters. The same is true for pharmacy technicians. Both pharmacists and technicians rarely discuss risk factors for falling with patients.

### In-depth perspectives (qualitative data)

**Fig. 3:**
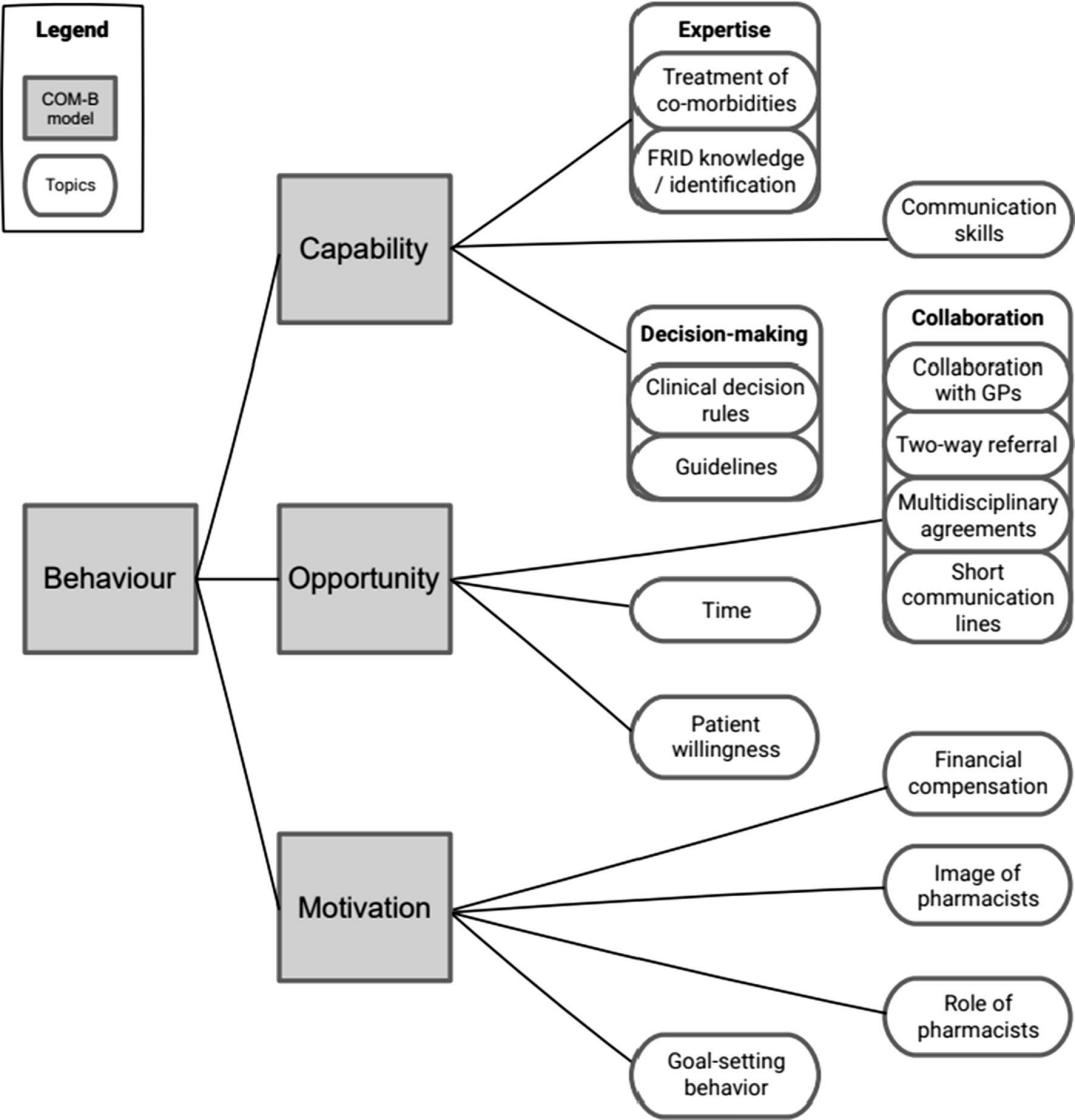
The topics of the interviews mapped to the domains of the capability opportunity motivation-behaviour (COM-B) model [28, 29]

Figure 3 illustrates the most important identified topics of the interviews and their mapping to the domains of the COM-B model. In Table 3, pharmacists’ quotes are related to the COM-B model and topics.

**Table 3:** Pharmacists’ quotes that describe the barriers and facilitators for implementing fall prevention care, linked to the topics, and the domains of the capability opportunity motivation-behaviour (COM-B) model

COM-B	Barrier/facilitator	Topic
Capability	Pharmacist 6, 59-year-old woman “Yes, it is always very complex, because most patients do not have just one problem but have 10 problems. These all intervene with each other. As a pharmacist, you try to determine, ‘what if we try this or this.’” *(barrier)*	Expertise: treatment of co-morbidities
	Pharmacist 10, 50-year-old man “When I provided the medication for a nursing home with elderly people, none of them was using antihypertensives anymore at a certain moment. […] These patients were sitting in their chair all day, had rarely any exercise, and deprescribing antihypertensives was necessary to prevent hypotension.” *(facilitator)*	Expertise: FRID recognition
	Pharmacist 8, 29-year-old woman “We worked hard for it. […]. We have always invested in the relationship. We have accessible contact. We use WhatsApp; we call; we see each other a lot. We organize meetings six times a year to make agreements about prescribing. And once a year, we have a social event.” *(facilitator)*	Collaboration: collaboration with GPs
	Pharmacist 3, 36-year-old woman “I think it is most important whether patient himself is willing. Patient motivation is most important, because otherwise you can change what you want, but maybe you create chaos and they lose their trust.” *(barrier)*	Patient willingness
	Pharmacist 16, 35-year-old woman “I used to go to the GP with all my suggestions based on clinical decision rules.” *(facilitator)*	Decision-making: clinical decision rules
	Pharmacist 7, 59-year-old man “I ask the patient, ‘When did you fall? At what time? How did it happen? Did you use medication and at what time?’ […] It is difficult, but I try to investigate whether there is a link with the medication or not. Sometimes you don’t know, because patients can also be dizzy because of other reasons.” *(facilitator)*	Decision-making
	Pharmacist 8, 29-year-old woman “After the Royal Dutch Pharmacists Association regional meeting, we decided to organize a fall prevention week. […] I am sure my pharmacy technicians are not regularly asking patients about fall history at the moment. I also doubt if they have the confidence to do that. So, before organizing this week, I will organize a training for the pharmacy technicians. Then they know what they are talking about and why it is important.” *(facilitator)*	Expertise
Opportunity	Pharmacist 12, 40-year-old woman “Yes, I think it is a missed opportunity [when GPs are not collaborating]. Not only for me, but for healthcare in general. […] I think we [pharmacists] are trained more by nature to look for collaboration opportunities. We are also more dependent, and GPs are often more autonomous.” *(barrier)*	Collaboration: collaboration with GPs
	Pharmacist 10, 50-year-old man [Replies to the observation that the collaboration seems to work well]: “Yes, that is in particular with the practice directly in the building. Outside it is a bit more difficult. Also there has been a switch of GPs working in the building. Some older GPs left and younger GPs came instead. I notice that there is lots of interest among younger GPs to work with us to improve healthcare for the patients.” *(facilitator)*	Collaboration: collaboration with GPs
	Pharmacist 4, 35-year-old woman “The physiotherapist already had a fall prevention programme […]. In the district we made agreements about it, and we receive money for the organization of this integrated primary care service.” *(facilitator)*	Collaboration: multidisciplinary agreements
	Pharmacist 2, 51-year-old woman “Sometimes the general practice nurse visits patients at home and finds all kinds of medication boxes. Well, nowadays we use ‘siilo-app’, that all healthcare providers use to communicate. Thus, then I receive a siilo-app in which the general practice nurse recommends me to visit the patient.” *(facilitator)*	Collaboration: short communication lines
	Pharmacist 11, 33-year-old woman “There are also a lot of older people who say, ‘I don’t want anything to change, suppose something happens to me.’” *(barrier)*	Patient willingness
Motivation	Pharmacist 5, 49-year-old man “When you want to make a good fall assessment, it takes a lot of time. This should not happen in the pharmacy, but together with the GP. Someone who can examine a bit more, like the stand-up-and-go test.” *(barrier)*	Identification of patients with high fall risk
	Pharmacist 1, 36-year-old man “I would recommend to colleagues to promote what you do and how you do it. For example, write an article in the neighbourhood newspaper about it.” *(facilitator)*	Image of pharmacists
	Pharmacist 15, 39-year-old woman “Well, the pharmacy technicians also need to do more with fall prevention. It would be nice if they could have some support, for example a course or information. Because at this moment, they are not going to ask about it, because they don’t know what to say when the patient answers.” *(barrier)*	Expertise
	Pharmacist 8, 29-year-old woman “I have doubts about if physicians are waiting for this [fall prevention]. But who knows, maybe we are able to present it sexy, and it will succeed. But we shall see then [at the multidisciplinary team meeting].” *(barrier)*	Collaboration: collaboration with GPs
	Pharmacist 8, 29-year-old woman “Fall prevention often means the withdrawal of drugs. And the business model of the pharmacy is based on the number of drugs dispensed. Thus, every drug you don’t dispense is a missed income.” *(barrier)*	Financial compensation
	Pharmacist 14, 38-year-old woman “In the regional elderly care work group, our pharmacists participate, which is a good thing. […] But I don’t really know what agreements they have made. So probably the agreements are not very concrete.” *(barrier)*	Goal-setting behaviour
	Pharmacist 1, 36-year-old man “For the withdrawal of antidepressants, we sent letters on 11–12–2019. Our aim was that 5% of the patients who received a letter would contact the GP or pharmacy within 3 months. However, this day (17–04–2020), we only received three replies. That is not much. We sent 126 letters.” *(facilitator)*	Goal-setting behaviour
	Pharmacist 2, 51-year-old woman “Deprescribing is also an ethical discussion. What happens when the blood pressure increases and someone gets hospitalized or dies and was not treated according to the guidelines.” *(barrier)*	Decision-making: guidelines
	Pharmacist 10, 50-year-old man “By asking patients about the consequences of falls. And let them realize that they lose confidence because of falls. That is very obvious: they become afraid when they have experienced a few falls, which impairs them. They go out less, become isolated. […] I make them realize that there could be a link with the medication use.” *(facilitator)*	Patient willingness
	Pharmacist 8, 29-year-old woman “At the moment, there will be financial compensation for fall prevention; I am sure every pharmacist will start with it.” *(facilitator)*	Financial compensation
	Pharmacist 11, 33-year-old woman “With the use of clinical decision rules these kinds of topics are also identified, and it increases your own knowledge.” *(facilitator)*	Decision-making: clinical decision rules
	Pharmacist 3, 36-year-old woman “Those patients just want to have their drugs on the bedside table. They need to take it for themselves. I am not going to change that. […] I ask everything out, and in case of the benefit of the doubt, I will let the lady keep using it.” *(barrier)*	Patient willingness

*GP* General practitioner

### Capability

In the interviews, pharmacists mentioned that their involvement in fall prevention should primarily cover the safe use of FRIDs in patients with high fall risk. Interviewed pharmacists mentioned that they are often unaware that patients have fallen, because patients do not report this.

#### Knowledge

In the interviews, all pharmacists mentioned that deprescribing is often possible. However, only a limited number of drugs are deprescribed easily, such as alpha-blockers for the treatment of benign prostatic hyperplasia. For most drugs, deprescribing is seen as a tedious process and pharmacists reported some knowledge gaps about FRIDs and limited proper deprescribing schemes.

#### Cognitive and interpersonal skills

Interviewed pharmacists considered the inability to convince patients about the relevance of deprescribing was seen as a major barrier, specifically for psychotropic drugs, including benzodiazepines. Pharmacists mentioned applying some effective strategies, such as the taper guidelines, encouraging patients to use benzodiazepines for only a short time, and sending letters to invite patients for consultation to support drug cessation. Effective communication skills are deemed necessary to motivate patients to cease benzodiazepines.

#### Decision-making

Pharmacists stated that the complexity of patients’ morbidities and drug treatment strongly influences decisions about deprescribing. They mentioned being unsure about the consequences for underlying treated diseases after deprescribing. Furthermore, they mentioned that both GPs and pharmacists prefer to carefully adjust medication in patients whose medication has been stable for a long-time.

Some pharmacists indicated that the pharmacy’s decision support systems sometimes facilitate the identification of medication-related problems. Pharmacists mentioned they wish for a clear decision-guiding overview of fall risk-increasing drugs on a set webpage. One pharmacist explained that in practice there is limited time to search for information in guidelines.

#### Multidisciplinary collaboration

Pharmacists mentioned that collaborative initiatives are helpful, such as regular reviews of older patients in multidisciplinary teams. Other healthcare providers sometimes initiate these collaborative fall prevention initiatives. For pharmacists, recognizing such initiatives is seen as important in order to join them. They stated that satisfying multidisciplinary collaboration is built through hard work, trust, and a time investment. Yet, pharmacists described experiencing some difficulties in collaboration, for example when they tried to convince GPs about the relevance of deprescribing in patients with high fall risk.

### Opportunity

#### Multidisciplinary collaboration and agreements

In interviews, pharmacists emphasized the importance of having agreements with healthcare providers concerning fall prevention. They mentioned that every healthcare provider’s role should ideally be captured in a fall prevention guideline. Regarding the collaboration with GPs, pharmacists mentioned that this collaboration is better organized with practices close to the pharmacy or in the same building. Pharmacists also reported that GPs are often reluctant to deprescribe, citing GPs’ dislike of time-consuming interventions and a potential lack knowledge on deprescribing as reasons.

Supportive infrastructure for referral and communication may help pharmacists to organize multidisciplinary fall prevention care. Some pharmacists proposed having short communication lines supporting referral from other healthcare providers to pharmacists, and vice versa.

#### Patient willingness and co-operation

Patient willingness to deprescribe medication was often mentioned as paramount for successful deprescribing. Moreover, interviewed pharmacists highlighted that this willingness to deprescribe FRIDs was dependent on the type of medication—patients were often unwilling to cease psychotropic drugs, benzodiazepines in particular.

Moreover, pharmacists stated that patients rarely report falls, do not relate medication use to their falls and seldom suggest medication deprescribing themselves. Pharmacists said that they only knew patients’ needs when they asked them directly. They also mentioned that patients could be afraid of medication deprescribing, for example, because their medication is stable, and they are afraid that modification will increase their morbidity risk.

### Motivation

#### Role and image of pharmacists

In the interviews, pharmacists mentioned that they see for themselves mainly a fall prevention role in the evaluation of FRID use. Some pharmacists reported they have the impression that their expertise in fall prevention, and especially in FRIDs is not always valued by both patients and GPs. Furthermore, these pharmacists feel they have to convince patients and GPs of this expertise. Many interviewed pharmacists lack concrete agreements with GPs about goals and each other’s role in fall prevention.

#### Identification of patients with high fall risk

Since medication reviews are a core business of pharmacists, this is seen as an important starting point for the identification of patients with high fall risk and the provision of fall prevention care. One interviewed pharmacist mentioned that pharmacists' accessibility to patients could facilitate the identification of patients with high fall risk.

#### Goal-setting behaviour

Few interviewed pharmacists set concrete goals for the provision of fall prevention, for example regarding the number of benzodiazepines that could be deprescribed annually. Furthermore, many interviewed pharmacists aim to provide a pre-determined number of medication reviews weekly or annually. They also evaluate whether they have reached these targets, which supported deprescribing.

#### Financial compensation

Some interviewed pharmacists believe that reimbursement is necessary as a motivator for pharmacists to implement fall prevention in daily practice.

## Discussion

In this mixed-methods study, we found that pharmacists are motivated to provide fall prevention services, but their capability differs. They have had diverse opportunities to provide fall prevention, with key facilitators being efficient collaboration and establishment of multidisciplinary agreements. Pharmacists indicated that major barriers were patient’s unwillingness to cease medication, the complexity of deprescribing, limited goal-setting behaviour, a lack of time, and a lack of financial compensation. It has previously been reported that pharmacists believe they should be involved in fall prevention; however, only a minority have actually been involved [24]. We showed similar results and gained insights in facilitators and barriers which are essential to know to foster further implementation of fall prevention.

Pharmacists believe they have the capability to contribute to fall prevention; in particular, they think their role in fall prevention should cover the monitoring of FRID use. Pharmacists mentioned that they already regularly suggest deprescribing of antihypertensives, antidepressants, and benzodiazepines. However, pharmacists reported that they did not always succeed in deprescribing FRIDs. In our study, barriers and facilitators for FRID deprescribing, including pharmacists being uncertain about harms and benefits of drug deprescribing, corresponded to barriers and facilitators in studies investigating deprescribing for other reasons [30]. Drug deprescribing could be facilitated by step-wise dose-reductions with in-between evaluations [30].

Deprescribing was perceived to be the most difficult for psychotropic drugs such as benzodiazepines. While pharmacists used a variety of communication skills to engage patients in FRID deprescribing, they reported that patients were often unwilling to cease benzodiazepines. Pharmacists could sometimes convince them by offering guidance and by increasing awareness about drug risks. Large variation in patients’ willingness to deprescribe drugs has previously been reported [31, 32]. For example, some patients owe their healthiness to their medication use and are, therefore, suspicious when it comes to deprescribing. An important aspect that facilitated patients’ decision-making in deprescribing was trust in their healthcare providers [31, 32].

Limited multidisciplinary collaboration, especially with GPs, was one of the most important barriers cited for the implementation of fall prevention in community pharmacies. This includes problems with convincing GPs about the importance, GPs having no time for pharmacists, and weak relationships with GPs from remote practices. Few pharmacists had multidisciplinary agreements about fall prevention. A lack of structured agreements regarding the referral of patients has previously been reported as a major barrier to pharmacists’ multidisciplinary collaboration, while pharmacists’ experience and confidence have been identified as facilitators for effective communication and collaboration with other healthcare providers [33]. In our study, pharmacists mentioned that efficient multidisciplinary care regarding fall prevention and deprescribing require hard work and a substantial time investment. Pharmacists had the impression that patients and other healthcare providers often did not clearly recognize the role of pharmacists in fall prevention. It has been reported previously that healthcare providers might even misunderstand pharmacists’ roles [33].

Lastly, pharmacists’ opportunity and motivation to provide fall prevention care were counteracted by a lack of both time and reimbursement. These findings correspond to previous findings emphasizing a need for reimbursement to motivate pharmacists to implement time-consuming pharmaceutical care interventions, such as fall prevention [24, 34].

### Strengths and weaknesses

The major strength of this study was the combination of both quantitative and qualitative methods for data collection, which enabled us to gain in-depth insight into the perspectives of pharmacists. We achieved sufficient participant response rates to the statements and the survey. These data were collected at the KNMP regional meetings, which were attended by diverse groups of Dutch community pharmacists. The demographics of the pharmacists who completed the survey and participated in the interviews correspond to those of the Dutch pharmacist population [35], thus implying that we were able to include a representative sample. Still, non-participating pharmacists who are not interested in fall prevention might be underrepresented and may hold other views and opinions. Therefore, community pharmacists’ motivation and capability to provide fall prevention services might be overestimated.

Another strength was the application of the COM-B model to interpret the qualitative data. The theoretical framework supported the identification of pharmacists’ needs to increase their capability, opportunity and motivation. For example, based on the findings from the COM-B, pharmacists thought they mainly require stronger opportunity [28, 29]. A limitation of the study was that only the analysis of the data and not the design was based on this theoretical framework. Data collection would presumably have been more targeted when the behavioural change theory was applied in advance, during the design of the study.

### Implications

First, pharmacists see improved multidisciplinary collaboration as a key facilitator for contributing effectively to fall prevention. In particular, multidisciplinary agreements should be formulated wherein the roles and tasks of pharmacists are stated. Overarching national agreements on pharmacists’ contribution to fall prevention would be supportive as well, and these individuals should ideally receive financial compensation for their contribution to fall prevention care. Second, pharmacists should demonstrate their motivation to participate in fall prevention care. They should define targets with regard to deprescribing, for example of benzodiazepines, to achieve success. Lastly, pharmacists could enhance their own capability by undertaking educational trainings, applying guidelines related to deprescribing, and becoming more experienced with providing fall prevention care. Additional clinical decision rules to support deprescribing may also facilitate fall prevention.

Future studies should investigate how pharmacists could improve multidisciplinary collaboration regarding fall prevention. Furthermore, actual implementation of fall prevention services in community pharmacies should be conducted and evaluated.

## Conclusion

Community pharmacists deem themselves capable of providing fall prevention services, and they are motivated to do so, particularly by deprescribing FRIDs. However, they perceive the decision-making of FRID deprescribing as complex due to the difficulties in weighing fall risk against treatment benefit for individual patients. Pharmacists believe they could provide better fall prevention services in collaboration with other disciplines.

## Supplementary Information

Below is the link to the electronic supplementary material.

Supplementary file1 (DOCX 24 kb)

## References

[1] Hopewell S, Adedire O, Copsey BJ, Boniface GJ, Sherrington C, Clemson L (2018). Multifactorial and multiple component interventions for preventing falls in older people living in the community. Cochrane Database Syst Rev..

[2] World Health Organization (2008). WHO global report on falls prevention in older age.

[3] Boyé ND, Van Lieshout EM, Van Beeck EF, Hartholt KA, Van der Cammen TJ, Patka P (2013). The impact of falls in the elderly. Trauma.

[4] Gillespie LD, Robertson MC, Gillespie WJ, Sherrington C, Gates S, Clemson LM (2012). Interventions for preventing falls in older people living in the community. Cochrane Database Syst Rev..

[5] de Vries M, Seppala LJ, Daams JG, van de Glind EMM, Masud T, van der Velde N (2018). Fall-risk-increasing drugs: a systematic review and meta-analysis: I. Cardiovascular drugs. J Am Med Dir Assoc..

[6] Seppala LJ, Wermelink AMAT, de Vries M, Ploegmakers KJ, van de Glind EMM, Daams JG (2018). Fall-risk-increasing drugs: a systematic review and meta-analysis: II. Psychotropics. J Am Med Dir Assoc.

[7] Seppala LJ, van de Glind EMM, Daams JG, Ploegmakers KJ, de Vries M, Wermelink AMAT (2018). Fall-risk-increasing drugs: a systematic review and meta-analysis: III. Others. J Am Med Dir Assoc.

[8] Karani MV, Haddad Y, Lee R (2016). The role of pharmacists in preventing falls among america’s older adults. Front Public Health.

[9] Stuart GM, Kale HL (2018). Fall prevention in central coast community pharmacies. Health Promot J Austr.

[10] Robinson JM, Renfro CP, Shockley SJ, Blalock SJ, Watkins AK, Ferreri SP (2019). Training and toolkit resources to support implementation of a community pharmacy fall prevention service. Pharmacy (Basel).

[11] Cooper JW, Burfield AH (2003). Medication interventions for fall prevention in the older adult. J Am Pharm Assoc.

[12] Walsh ME, Boland F, Moriarty F, Fahey T (2019). Modification of potentially inappropriate prescribing following fall-related hospitalizations in older adults. Drugs Aging.

[13] Blalock SJ, Casteel C, Roth MT, Ferreri S, Demby KB, Shankar V (2010). Impact of enhanced pharmacologic care on the prevention of falls: a randomized controlled trial. Am J Geriatr Pharmacother.

[14] van der Velde N, Stricker BHC, Pols HAP, van der Cammen TJM (2007). Risk of falls after withdrawal of fall-risk-increasing drugs: a prospective cohort study. Br J Clin Pharmacol.

[15] Verdoorn S, Kwint H-F, Blom J, Gussekloo J, Bouvy ML (2018). DREAMeR: Drug use Reconsidered in the elderly using goal attainment scales during medication review; study protocol of a randomised controlled trial. BMC Geriatr.

[16] Verdoorn S, Kwint H-F, Blom JW, Gussekloo J, Bouvy ML (2019). Effects of a clinical medication review focused on personal goals, quality of life, and health problems in older persons with polypharmacy: a randomised controlled trial (DREAMeR-study). PLoS Med..

[17] Perell KL, Manzano MLP, Weaver R, Fiuzat M, Voss-McCarthy M, Opava-Rutter D (2006). Outcomes of a consult fall prevention screening clinic. Am J Phys Med Rehabil.

[18] Shahsavari H, Matourypour P, Ghiyasvandian S, Nejad MRG (2020). Medical Research Council framework for development and evaluation of complex interventions: a comprehensive guidance. J Educ Health Promot.

[19] Child S, Goodwin V, Garside R, Jones-Hughes T, Boddy K, Stein K (2012). Factors influencing the implementation of fall-prevention programmes: a systematic review and synthesis of qualitative studies. Implement Sci.

[20] van Rhyn B, Barwick A (2019). Health practitioners’ perceptions of falls and fall prevention in older people: a metasynthesis. Qual Health Res.

[21] Liddle J, Lovarini M, Clemson L, Mackenzie L, Tan A, Pit SW (2018). Making fall prevention routine in primary care practice: perspectives of allied health professionals. BMC Health Serv Res.

[22] Vlaeyen E, Stas J, Leysens G, Van der Elst E, Janssens E, Dejaeger E (2017). Implementation of fall prevention in residential care facilities: A systematic review of barriers and facilitators. Int J Nurs Stud.

[23] Casserlie LM, Mager NAD (2016). Pharmacists’ perceptions of advancing public health priorities through medication therapy management. Pharm Pract (Granada).

[24] Laliberté M-C, Perreault S, Damestoy N, Lalonde L (2013). The role of community pharmacists in the prevention and management of osteoporosis and the risk of falls: results of a cross-sectional study and qualitative interviews. Osteoporos Int.

[25] Garcia-Cardenas V, Rossing CV, Fernandez-Llimos F, Schulz M, Tsuyuki R, Bugnon O (2020). Pharmacy practice research: a call to action. Res Social Adm Pharm.

[26] Tong A, Sainsbury P, Craig J (2007). Consolidated criteria for reporting qualitative research (COREQ): a 32-item checklist for interviews and focus groups. Int J Qual Health Care.

[27] Michie S, van Stralen MM, West R (2011). The behaviour change wheel: a new method for characterising and designing behaviour change interventions. Implement Sci.

[28] Michie S, Atkins L, West R (2014). The behaviour change wheel: a guide to designing interventions.

[29] Cowdell F, Dyson J (2019). How is the theoretical domains framework applied to developing health behaviour interventions? A systematic search and narrative synthesis. BMC Public Health.

[30] Anderson K, Foster M, Freeman C, Luetsch K, Scott I (2017). Negotiating, “unmeasurable harm and benefit”: perspectives of general practitioners and consultant pharmacists on deprescribing in the primary care setting. Qual Health Res.

[31] Weir K, Nickel B, Naganathan V, Bonner C, McCaffery K, Carter SM (2018). Decision-making preferences and deprescribing: perspectives of older adults and companions about their medicines. J Gerontol B Psychol Sci Soc Sci.

[32] Crutzen S, Baas G, Abou J, van den Born-Bondt T, Hugtenburg JG, Bouvy ML (2020). Barriers and enablers of older patients to deprescribing of cardiometabolic medication: a focus group study. Front Pharmacol.

[33] Sim TF, Hattingh HL, Sunderland B, Czarniak P (2020). Effective communication and collaboration with health professionals: a qualitative study of primary care pharmacists in Western Australia. PLOS ONE.

[34] Ensing HT, Koster ES, Sontoredjo TAA, van Dooren AA, Bouvy ML (2017). Pharmacists’ barriers and facilitators on implementing a post-discharge home visit. Res Soc Admin Pharm..

[35] Farmaceutische SK (2015). Openbaar apotheker wordt vrouwenberoep. Pharm Weekbl.

