# Supplementary material for: Community pharmacists’ perceptions on providing fall prevention services: a mixed-methods study
Source: Int J Clin Pharm. 2021 Jun 13;43(6):1533–45. doi: 10.1007/s11096-021-01277-4 (PMC8642357; doi:10.1007/s11096-021-01277-4)
Supplement: Supplementary file 1 — Supplementary file1 (DOCX 24 kb) [file 11096_2021_1277_MOESM1_ESM.docx]

# Supplementary Information (SI)

**Fall prevention and deprescribing of fall risk-increasing drugs: the community pharmacists’ perspective**

Marle Gemmeke MSc PharmD^1^, Ellen S. Koster PhD^1^, Eline A. Rodijk BSc^1^, Katja Taxis PhD PharmD ^2^, Marcel L. Bouvy PhD PharmD^1^

^1^Division of Pharmacoepidemiology and Clinical Pharmacology, Utrecht Institute for Pharmaceutical Sciences (UIPS), Faculty of Science, Utrecht University, Utrecht, The Netherlands
^2^Department of Pharmacotherapy, Pharmacoepidemiology and Pharmacoeconomics (PTEE), Faculty of Science and Engineering, Groningen Research Institute of Pharmacy, University of Groningen, Groningen, The Netherlands

**Journal:**

International Journal of Clinical Pharmacy

**Corresponding author:**

E.S. (Ellen) Koster, PhD

Division of Pharmacoepidemiology and Clinical Pharmacology

Utrecht Institute for Pharmaceutical Sciences

Faculty of Science, Utrecht University

PO Box 80082, 3508 TB, Utrecht, The Netherlands

Tel: +31 (0)30 253 7324 / Fax: +31 (0)30 253 9166 / Email: [E.Koster@uu.nl](mailto:E.Koster@uu.nl)

# S1: Consolidated criteria for reporting qualitative studies (COREQ): 32-item checklist

*Table 1. Consolidated criteria for reporting qualitative studies (COREQ): 32-item checklist*

| **No** | **Item** | **Guide questions/description** | **Check?** |
| --- | --- | --- | --- |
| **Domain 1: Research team and reflexivity** | | | |
| **Personal Characteristics** | | | |
| **1.** | Interviewer/  facilitator | Which author/s conducted the interview or focus group? | The interviews were conducted by a Master student-researcher (ER). |
| **2.** | Credentials | What were the researcher's credentials? E.g. PhD, MD | Data were collected by Master student-researcher ER. Data were analysed by postgraduate student researcher MG. Analyses were checked, reviewed and supervised by experienced postgraduate researchers EK, KT and MB. |
| **3.** | Occupation | What was their occupation at the time of the study? | ER was a Master Pharmacy student. MG was a postgraduate student researcher. EK, KT and MB were experienced postgraduate researchers. |
| **4.** | Gender | Was the researcher male or female? | ER was female. |
| **5.** | Experience and training | What experience or training did the researcher have? | ER had little experience with interviewing, since she completed a Master pharmacy course in qualitative research. She was supervised by MG and EK. EK was experienced with performing qualitative research. |
| **Relationship with participants** | | | |
| **6.** | Relationship established | Was a relationship established prior to study commencement? | At the KNMP regional meetings ER met few pharmacists who participated in the interviews afterwards, but no strong relationship was already built. |
| **7.** | Participant knowledge the interviewer | What did the participants know of about the researcher? e.g. personal goals, reasons for doing the research | The participants were informed about the research by the presentation during the KNMP regional meeting, by completing the survey and received an invitation e-mail with information about the topics of the interview. |
| **8.** | Interviewer characteristics | What characteristics were reported about the interviewer/facilitator? e.g. Bias, assumptions, reasons and interests in the research topic | The interviewer was a Master Pharmacy student and her main interest was to investigate the needs and wants of pharmacists with regard to deprescribing of FRIDs and multidisciplinary collaboration. |
| **Domain 2: study design** | | | |
| **Theoretical framework** | | | |
| **9.** | Methodological orientation and Theory | What methodological orientation was stated to underpin the study? e.g. grounded theory, discourse analysis, ethnography, phenomenology, content analysis | The COM-B system and TDF were used to analyse the data and underpin the study. |
| **Participant selection** | | | |
| **10.** | Sampling | How were participants selected? e.g. purposive, convenience, consecutive, snowball | Participants could sign up for an interview by the use of a reply coupon in the survey. |
| **11.** | Method of approach | How were participants approached? e.g. face-to-face, telephone, mail, email | Participants were approached by e-mail. The interview was either by telephone or video-call. |
| **12.** | Sample size | How many participants were in the study? | 16 pharmacists were interviewed. |
| **13.** | Non-participation | How many people refused to participate or dropped out? Reasons? | 19 pharmacists signed up for the interview but did not participate eventually. |
| **Setting** | | | |
| **14.** | Setting of data collection | Where was the data collected? *e*.g. home, clinic, workplace | Pharmacists were either at their workplace or at home. The interviewer was at her home. |
| **15.** | Presence of non-participants | Was anyone else present besides the participants and researchers? | No, but pharmacists could be interrupted by questions of their technicians. |
| **16.** | Description of sample | What are the important characteristics of the sample? e.g. demographic data, date | Relevant background characteristics of pharmacists were described in Figure 2. |
| **Data collection** | | | |
| **17.** | Interview guide | Were questions, prompts, guides provided by the authors? Was it pilot tested? | The questions, prompts and guides were provided by the authors, but not pilot tested. |
| **18.** | Repeat interviews | Were repeat interviews carried out? If yes, how many? | There were no repeat interviews. |
| **19.** | Audio/visual recording | Did the research use audio or visual recording to collect the data? | The interviewer used audio-recording during the interviews. |
| **20.** | Field notes | Were field notes made during and/or after the interview or focus group? | Field notes were made during the interview. The interview was transcribed verbatim directly afterwards. |
| **21.** | Duration | What was the duration of the interviews or focus group? | The durations of the interviews were 20-35 minutes. |
| **22.** | Data saturation | Was data saturation discussed? | Data saturation was discussed after 16 interviews. |
| **23.** | Transcripts returned | Were transcripts returned to participants for comment and/or correction? | Pharmacists were offered to request their transcript. One pharmacist requested the transcript, but had no comments. A summary of the findings of the interviews was returned to participants by publication in a national pharmacy magazine. |
| **Domain 3: analysis and findings** | | | |
| **Data analysis** | | | |
| **24.** | Number of data coders | How many data coders coded the data? | The data was coded by one researcher (MG) and the linking of quotes to the TDF domains was reviewed by two researchers (EK, MB) |
| **25.** | Description of the coding tree | Did authors provide a description of the coding tree? | The topics are found in Appendix S1. The data was analysed using the COM-B system and TDF. |
| **26.** | Derivation of themes | Were themes identified in advance or derived from the data? | The topic list was prepared in advance. The COM-B system and TDF were applied during data analysis. |
| **27.** | Software | What software, if applicable, was used to manage the data? | NVivo version 12 software was used to analyse the data. |
| **28.** | Participant checking | Did participants provide feedback on the findings? | Participant did not provide feedback on the findings. |
| **Reporting** | | | |
| **29.** | Quotations presented | Were participant quotations presented to illustrate the themes / findings? Was each quotation identified? e.g. participant number | Participant quotations were presented to illustrate the findings. All quotations were identified by participation number. |
| **30.** | Data and findings consistent | Was there consistency between the data presented and the findings? | The research theme believes there was consistency between the presented data and the findings. |
| **31.** | Clarity of major themes | Were major themes clearly presented in the findings? | The COM-B system and TDF were used during data analyses to present the themes clearly. |
| **32.** | Clarity of minor themes | Is there a description of diverse cases or discussion of minor themes? | Diverse cases were described and also minor themes (e.g. opinions of single participants) were mentioned. |

*Abbreviations: FRID = fall risk-increasing drug, KNMP = Royal Dutch Pharmacists Association, COM-B system = Capability Opportunity Motivation – Behaviour system, TDF = Theoretical Domains Framework*

# S2: Questions addressed in the presentation and survey, and topics addressed in the interviews

*Table 2. Questions/statements addressed in the presentation and survey, and topics addressed in the interviews.*

| ***Lecture*** | ***Survey*** | | ***Interviews*** |
| --- | --- | --- | --- |
| **Statements**  **(Likert scale disagree (0) to agree (10))** | **Statements**  **(Likert scale never (1) to always (5))** | I experience difficulties with starting a conversation with patients about the effects of their medication use on their fall risk | **Topics** |
| Community pharmacists can contribute to fall prevention | When I perform a medication review, I suggest medication modifications if I know the patient has fall experiences | I need a guideline that supports me with deprescribing FRIDs | ● Role of pharmacists |
| I have enough knowledge to recognize FRIDs | I ask about fall history when I perform a medication review | A guideline that supports me to deprescribe FRIDs is not going to help me, because deprescribing should be tailored to individual patient circumstances | ● Current contribution and activities |
| I have the capabilities to recognize patients with high fall risk | Proactively I ask patients about fall history (at the counter or in a telephone call) | I am going to spend more time and attention to fall prevention in my daily practice | ● Capabilities of pharmacists |
| At the moment I contribute to fall prevention | The pharmacy technicians proactively ask patients about fall history |  | ● Knowledge |
| I discuss fall prevention at medication reviews | I discuss with patients their risk factors for falling | **Questions** | ● Needs of pharmacists |
| Beyond medication reviews I discuss fall prevention | The pharmacy technicians discuss patients’ risk factors for falling with them | Do you have multidisciplinary agreements about fall prevention? (Yes/No) | ● Multidisciplinary collaboration |
| I have enough time to organize fall prevention care |  | If you have multidisciplinary agreements about fall prevention, with whom? (▫general practitioner, ▫physiotherapist, ▫home care, ▫nursing home physician, ▫dietician, ▫geriatrician, ▫other) |  |
| Recognizing patients with fall risk belongs to one of the tasks of community pharmacists | **Statements**  **(Likert scale disagree (1) to agree (5))** | What are your needs to be able to do more in fall prevention? (▫multidisciplinary collaboration, ▫reimbursement, ▫time, ▫training for pharmacy technicians, ▫patient information material, ▫guideline to deprescribe FRIDS, ▫more knowledge/training) |  |
| Fall prevention care belongs to tasks of community pharmacists | I experience difficulties with the recognition of patients with high fall risk |  |  |
